# Brain abscess following acute otitis media in a nasopharyngeal carcinoma survivor: a case report

**DOI:** 10.1097/RC9.0000000000000123

**Published:** 2026-02-25

**Authors:** Kaori Shijo, Takashi Anzai, Yusuke Takata, Masahiro Nakamura, Satoshi Hara, Fumihiko Matsumoto

**Affiliations:** Department of Otorhinolaryngology, Juntendo University Faculty of Medicine, Tokyo, Japan

**Keywords:** acute otitis media, brain abscess, chemoradiotherapy, household transmission, nasopharyngeal carcinoma

## Abstract

**Introduction:**

Although rare, brain abscesses secondary to acute otitis media (AOM) may pose serious risks, particularly among adolescent and young adult (AYA) survivors of head and neck cancer.

**Presentation of case:**

A 29-year-old woman who survived AYA nasopharyngeal carcinoma following chemoradiotherapy developed an AOM complicated by a left temporal lobe brain abscess and motor aphasia. After undergoing neurosurgical drainage and receiving intravenous antibiotics, she achieved full recovery without mastoidectomy.

**Discussion:**

Eustachian-tube dysfunction resulting from prior radiotherapy and household exposure to respiratory pathogens were identified as possible contributing factors. This case illustrates the susceptibility of AYA cancer survivors to severe otogenic infections due to treatment-related sequelae.

**Conclusion:**

Clinicians should maintain vigilance for intracranial complications of AOM in AYA patients with a history of head and neck cancer therapy.

## Introduction

Brain abscesses are a life-threatening yet exceedingly rare complication of acute otitis media (AOM)^[^1^]^. Although uncommon in healthy individuals, survivors of head and neck cancer, especially those treated for nasopharyngeal carcinoma (NPC), may experience anatomical and mucosal alterations after radiotherapy, including Eustachian-tube dysfunction and impaired middle-ear ventilation, which predispose them to otologic infections^[^2^]^. NPC can develop in adolescents and young adults (AYAs) aged 15–39 years who often assume childcare responsibilities. As young children are reservoirs of respiratory pathogens, adult caregivers experience increased exposure to community-acquired viral and bacterial infections within households^[^3^]^. This case suggests that pathogen exposure and post-treatment anatomical susceptibility may predispose NPC survivors to serious complications from common infections.

**HIGHLIGHTS**

- Acute otitis media (AOM) complicated by temporal lobe brain abscess is a rare but life-threatening condition.
- The patient was a young female nasopharyngeal carcinoma survivor previously treated with chemoradiotherapy.
- Eustachian tube dysfunction due to prior therapy and household exposure to respiratory pathogens were key risk factors.
- This case underscores the need for early recognition and intervention in AYA cancer survivors at risk of intracranial complications from AOM.

## Presentation of case

**Figure 1.**
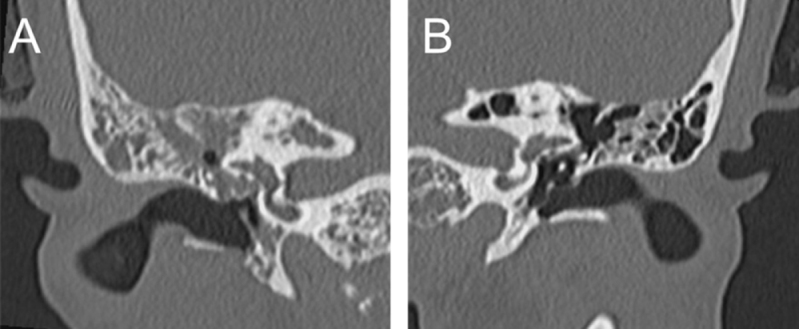
Axial temporal bone computed tomography. (A) Right side: Soft tissue opacity was observed in the tympanic and mastoid cavities. (B) Left side: Adequate aeration was preserved in the tympanic and mastoid cavities.

A 29-year-old woman who underwent chemoradiotherapy for NPC at age 16 presented with a 2-week history of bilateral otalgia, right-sided otorrhea, and persistent fever. She was raising two preschool-aged children at home who presented with symptoms consistent with sinusitis and otitis media during the same season. Despite receiving prior treatment for presumed AOM, her symptoms failed to improve. Otoscopic examination revealed right tympanic membrane perforation with purulent discharge and left tympanic membrane bulging. No findings suggested NPC recurrence. Temporal bone computed tomography (CT) showed soft tissue opacities in the right tympanic cavity and mastoid air cells (Fig. 1A), a well-aerated left ear (Fig. 1B), and no intracranial abnormalities. A diagnosis of severe AOM was established, and left myringotomy was performed. After obtaining an otorrhea sample for culture, oral lascufloxacin and 1.5% levofloxacin ear drops were initiated.

**Figure 2.**
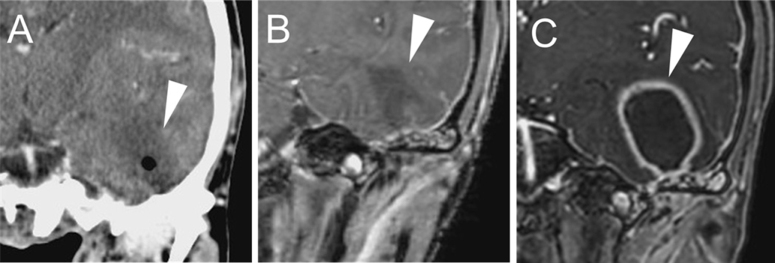
Imaging findings of the left temporal lobe. (A) Follow-up computed tomography (day 1 of hospitalization): Intracranial gas was observed. (B) Follow-up magnetic resonance imaging (MRI; day 1 of hospitalization): A low-intensity area was observed on T2-weighted imaging. (C) Follow-up MRI (day 18 of hospitalization): Abscess formation was observed.

On day 5 after the first visit, the patient developed worsening otalgia, fever, headache, and signs suggestive of motor aphasia. Contrast-enhanced head CT revealed low-density areas in the left tympanic cavity and mastoid air cells, along with intracerebral gas (Fig. 2A).

Brain magnetic resonance imaging (MRI) showed diffuse dural thickening with contrast enhancement over the left hemisphere. In the left temporal lobe, a 4.4-cm, T1-hypointense, contrast-enhancing lesion with surrounding vasogenic edema was observed, consistent with meningoencephalitis and suspected abscess formation (Fig. 2B).

Laboratory blood tests showed a normal white blood cell (WBC) count (8.3 × 10^9^/l; reference range, 3.6–8.9 × 10^9^/l) but a markedly elevated C-reactive protein (CRP) level (25.36 mg/dl; reference range, < 0.3 mg/dl), indicating significant inflammatory response. Cerebrospinal fluid analysis revealed a high WBC count (127/μl; reference range, 0–5 μl), elevated total protein (70 mg/dl; reference range, 10–40 mg/dl), and decreased glucose level (2.49 mmol/l; reference range, 2.78–4.16 mmol/l), suggesting meningitis. Pure-tone audiometry revealed mixed hearing loss, with thresholds of 62.5 and 47.5 dB in the right and left ears, respectively (normal hearing was defined as <25 dB HL according to the World Health Organization classification). No associated vertigo or nystagmus was observed.

After the otorrhea bacterial culture showed group A β-hemolytic *Streptococcus*, a diagnosis of streptococcal otogenic brain abscess secondary to AOM was established. Intravenous meropenem and vancomycin were promptly initiated.

On day 4, white purulent discharge was observed from the left tympanic membrane, which increased with the Valsalva maneuver. Although cerebrospinal fluid leakage was suspected, transferrin testing of the otorrhea was negative.

After 1 week of treatment, inflammatory markers (WBC: 8400/μl; CRP: 1.30, 61 mg/dl) improved markedly, along with language disturbance resolution. Based on antimicrobial susceptibility results, antibiotic therapy was deescalated to ceftriaxone to narrow the spectrum while maintaining effective coverage.

However, follow-up contrast-enhanced MRI on day 18 revealed abscess cavity enlargement in the left temporal lobe (Fig. 2C). On day 20, burr hole drainage performed by the neurosurgical team, and antibiotic therapy was re-escalated to meropenem and vancomycin, which significantly improved her otoscopic findings within 2 weeks. Despite the decrease in abscess cavity size, surrounding vasogenic edema increased. Bacterial culture of the drained material came back negative; however, metronidazole was added for potential anaerobic pathogens due to the persistent edema.

MRI 1 month after drainage showed only postoperative changes, with complete resolution of the abscess and perilesional edema. Intravenous antibiotics were administered for 60 days, and the patient was discharged on day 67.

At discharge, a large perforation remained in the right tympanic membrane, whereas the left tympanic membrane had normalized. Although audiometry demonstrated a 16.3-dB improvement in the left-sided hearing threshold, right-sided conductive hearing loss persisted due to the tympanic membrane perforation.

## Discussion

This case underscores the potential severity of AOM complications in patients with anatomical vulnerabilities. Our patient developed a brain abscess in the left temporal lobe, causing transient motor aphasia. Although rare, the occurrence of speech disturbances with systemic infection and ear pathology should prompt consideration of cortical involvement.

Intracranial complications secondary to otitis media are life-threatening and often present with otorrhea, headaches, neurological symptoms^[^4^]^. Several studies have described the development of brain abscesses following radiotherapy for NPC^[^2,5,6^]^, subsequently identifying structural skull-base vulnerability and concurrent otologic infections as plausible contributors, which is consistent with the present case. Radiotherapy for NPC has been associated with multiple late injuries involving the temporal bone and skull base, with consequent functional impairment. Radiation induces fibrosis of the tensor veli palatini and impairs Eustachian-tube ciliary function, causing ventilatory dysfunction^[^7^]^. In radiation-induced temporal lobe necrosis, small-vessel coagulative and fibrinoid necrosis induces parenchymal ischemia, with the infarcted brain being prone to abscess formation^[^8^]^. Moreover, microvascular injury and altered bone metabolism contribute to skull-base necrosis^[^9^]^. A recent the meta-analysis showed that approximately 3% of otogenic brain abscesses were related to AOM^[^4^]^.

Given that young children are a significant source of respiratory pathogens in households, adult contact with children increases their risk of respiratory tract infections^[^3^]^. Although this finding does not directly link household exposure to adult-onset AOM, it supports possible contribution of frequent upper respiratory infections in our case.

Adult-onset AOM typically requires prolonged intravenous antibiotics and neurosurgical and/or otologic drainage^[^4^]^. Importantly, our patient showed a significant improvement in both the brain abscess and middle-ear pathology following neurosurgical drainage and intravenous antibiotics, which prompted us to reconsider mastoidectomy. However, this favorable outcome reflects the specific course in our patient. Mastoidectomy or other otologic surgical interventions remain essential when middle-ear disease does not resolve or infection control is insufficient^[^10^]^.

Despite their unique lifestyle-related exposures, AYA survivors are often overlooked in infectious risk stratification. This case illustrates that pediatric respiratory illnesses can be a critical source of infection among vulnerable adults and that urgent neuroimaging should be considered when symptoms such as aphasia arise.

Although this case report presents important insights, caution should be exercised when interpreting or generalizing our findings.

## Limitations

Although this case report presents important insights, the present findings should be interpreted with caution, and generalization of these findings requires accumulation of further cases

## Conclusion

Brain abscesses can be a severe complication of AOM in patients treated for head and neck cancer. Clinicians should remain vigilant in recognizing the unique risk profile of AYA survivors, particularly those with young children at home. Early imaging and prompt interventions are essential to prevent morbidity.

## SCARE guidelines

This work has been reported in accordance with the SCARE criteria (Surgical Case Report Guidelines)^[^11^]^.
